# Heavy metals in leathers, artificial leathers, and textiles in the context of quality and safety of use

**DOI:** 10.1038/s41598-022-08911-9

**Published:** 2022-03-24

**Authors:** Elżbieta Bielak, Ewa Marcinkowska

**Affiliations:** Department of Quality and Safety of Non-food Products, Institute of Quality Sciences and Product Management, Cracow University of Economics, Rakowicka 27, 31-510 Cracow, Poland

**Keywords:** Health care, Risk factors, Materials science

## Abstract

The article presents research findings on the content of arsenic, cadmium, chromium, copper, lead, and zinc in extracts from leathers, artificial leathers intended for footwear components, and textiles. After extracting the metals using an artificial acidic sweat solution, their contents were quantitatively determined by atomic absorption spectrometry. In the cotton textiles, the metal contents were in accordance with the OEKO-TEX limits, while regarding the artificial leathers, only the acrylic knit fur had a too high chromium content (1.1 mg/kg) as compared with the requirements of the *STANDARD 100 by OEKO-TEX* for products intended for children (< 1.0 mg/kg). The chromium content in lining and upper leather (> 228.0 mg/kg) exceeds the limits for children’s products (< 2.0 mg/kg), but also the less restrictive ones for other products (< 200.0 mg/kg). Regarding the other metals, the leathers met the OEKO-TEX requirements. Approved materials may have elevated heavy metal contents, as demonstrated for chromium. The presence of heavy metals in too large amounts in products is a serious problem due to their allergenic and toxic effect. Therefore, action should be taken aimed at more effective detection and elimination of such products from markets and at reducing the use of chemicals containing harmful metals in manufacturing processes.

## Introduction

The basic materials used in the clothing and footwear industry include textiles, leathers, and artificial leathers, i.e., synthetic materials imitating natural leather, which are most often obtained by applying a layer of plastic (e.g., polyvinyl chloride) onto a textile carrier (e.g., fabric) or by impregnating a textile carrier with synthetic resins (e.g., polyurethane) and resin coagulation in the carrier. The presence of harmful substances in too large amounts in materials may pose a threat to the consumer’s health, especially in relation to products intended for children, as well as other products that are in direct contact with human skin. Heavy metals are an example. The mechanism of the toxic effect of metals and semimetals, the symptoms of poisoning, as well as the long-term effects vary. In the case of arsenic (As), skin lesions are the most characteristic symptom of the poisoning resulting from long-term exposure. The aforementioned semimetal damages the liver and hearing^1^; it is also believed to have a carcinogenic effect (including cancers of the skin, liver, prostate and Kupffer cell)^2^. The long-term inhalation of cadmium (Cd) vapour leads to chronic bronchitis, emphysema and even lung cancer^3^. Exposure to this metal leads to damage of the glomeruli and proximal tubules^4^. Contact with chromium (Cr) can contribute to the occurrence of eczema and allergies. In chronic poisoning (occupational exposure), changes in the kidneys, digestive track and circulatory system can be observed^1^. Skin itching and dermatitis can result from the exposure to copper (Cu) compounds, which are also responsible for conjunctivitis, ulceration and clouding of the cornea or throat, as well as damaging liver functions^5^. A characteristic symptom of lead (Pb) toxicity to the organism is anaemia, more common for children than adults. Apart from the disorders of the hematopoietic system, this metal is responsible for damage to the nervous system and has a negative effect on the digestive system and kidneys^5^, and chronic exposure to Pb leads to their failure^3^. One of the most important causes of zinc (Zn) toxicity for humans and animals are probable disturbances in the metabolism of metals that are necessary for the human body^5^, e.g., an oversupply of Zn leads to a decrease in iron absorption and, at the same time, an increase in the excretion of this element from the organism^6^.

The presence of heavy metals in materials should be considered both in terms of harmful effects on human health (related not only to the use thereof, but also to the production processes), but also on the environment, i.e., water, soil, and air, which they penetrate with, for example, wastewater from tanneries or factories where textiles are manufactured. This problem is very urgent and is widely analysed in many research works^7–15^. Many national and international organisations have developed regulations and guidelines on the permissible content of heavy metals in water, air, and food. For instance, according to the World Health Organisation, guideline values that are of health significance in drinking water for As are 0.01 mg/L; Cd—0.003 mg/L, Cr—0.05 mg/L, Cu—2 mg/L, and Pb—0.01 mg/L, Zn—not of health concern at levels found in drinking water^16^.

Due to the considerable amount of various harmful chemicals used in connection with the production processes of clothing and footwear materials as well as the pollutants contained in them, which are a burden to the environment, efforts are constantly being made to eliminate them using improved or completely new solutions and technologies. Bagheri et al.^17^ presented the option of using covalent organic frameworks (COFs) as sorbents and catalysts useful in removing pollutants such as heavy metals or dyes. Experiments prove that synthetic textile dyes, due to their toxic and genotoxic effects, are a threat to aquatic organisms and their habitats^18^. A very good efficacy (over 98%) of a hybrid material obtained by reacting biomass Ayous wood sawdust, with 3-aminopropyl-triethoxysilane as an adsorbent to remove the Reactive Blue 4 textile dye from wastewater effluents was demonstrated by Teixeira et al.^19^. An important problem in the context of pollution of water bodies is the increasing amount of micropollutants, such as organic polymers or suspended solids. It is possible to remove them from wastewater in an effective and environmentally safe manner by using powdered activated carbon^20^. Furthermore, Rasheed et al.^21^ proposed photocatalytic and adsorptive remediation of hazardous pollutants using a combination of metal–organic frameworks (MOFs) with nano-carbon materials. For example, scientists confirmed the possibility of using adsorbents to remove pollutants such as heavy metals, volatile organic compounds, and toxic gases from the atmospheric environment. It should be noted that an improvement in air quality has been observed recently because of the measures taken by governments of individual countries to counter the spread of the coronavirus pandemic. Globally, a decrease of carbon emissions has been observed, which has been linked to, among others, a reduction of activities in many industries and transportation^22^.

The number of scientific reports from recent years concerning studies on the content of heavy metals in natural leathers and textiles is limited, and concerning synthetic materials such as artificial leather, this number is negligible. Likewise, there are only a limited number of studies where the heavy metal content was determined simultaneously in different types of materials according to a standardised procedure, using a single measurement method, which would make it possible to conduct comparative analyses of the results. In 2015, Zhao et al.^23^ carried out experiments to compare two methods for determining the contents of Cd, Co, Cr, Cu, Hg, Ni and Pb in leathers and furs, i.e., the suggested microwave plasma-atomic emission spectrometry (MP-AES) method and the standardised inductively coupled plasma-atomic emission spectrometry (ICP-AES) method. The content of the same metals and additionally Al and Zn in natural leathers and artificial leathers for upholstery using inductively coupled plasma optical emission spectrometry (ICP-OES) was also determined by Aslan and Üzüm in 2015^24^. Three years earlier, Yaşa et al.^25^ also used the ICP-OES method to investigate the contents of Cd, Co, Cr, Cu, Ni, Pb and Zn in natural leathers for footwear uppers and lining while in 2011, Karavana et al.^26^ detected the presence of As, Ba, Cd, Cr, Hg, Pb, Sb and Se in sheepskins for children’s footwear. Due to the extensive contact with the body, leathers used for insoles and shoe uppers and clothing were tested in 2006 in Turkey for Co, Cr, Cu, Pb, Ni and Zn using ICP-AES^27^. In 2007 Rezić and Steffan^28^ analysed the contents among others of Al, As, Cd, Cr, Cu, Mn, Ni and Zn in textiles from Croatia using ICP-OES.

In Turkey, in 2015, different types of fibres used in the textile industry, i.e., cotton, acrylic, polyester, nylon, viscose, and polypropylene, were tested for the presence of Al, Cd, Co, Cr, Cu, Fe, Mn, Ni, Pb, Tl and Zn by MP-AES^29^, while in Italy, in 2009, Cr was detected in a wool top using atomic absorption spectrometry (AAS)^30^. In Turkey, in 2008, the contents of Cd, Cu, Fe, Mn, Ni and Zn in textile samples were determined also using AAS^31^, while in 2004, in Poland, the contents of Cd, Co, Cr, Cu, Ni, Pb and Zn in cotton bed cloth was analysed using ICP^32^.

The study aimed to determine whether randomly selected leathers and artificial leathers, approved for marketing and intended for the inside components of footwear and uppers, as well as textiles used in the production of children’s clothes, meet the requirements of the *STANDARD 100 by OEKO-TEX*^33^ and the *LEATHER STANDARD by OEKO-TEX*^34^ regarding the permissible content of metals in leathers and textiles. The quantitative determination of the content of arsenic, cadmium, chromium, copper, lead and zinc in materials, using the method of atomic absorption spectrometry, was preceded by extraction of the metals from the samples with artificial acidic sweat solution. The study's novelty concerns the risk assessment related to the presence of harmful metals in leathers, artificial leathers and textiles produced and/or available in the Polish market. The results may provide a starting point for further work aimed at identifying and verifying the hazards associated with the presence of harmful chemicals in products approved for marketing, as well as for taking action to eliminate them.

## Materials and methods

### Research material

**Figure 1:**
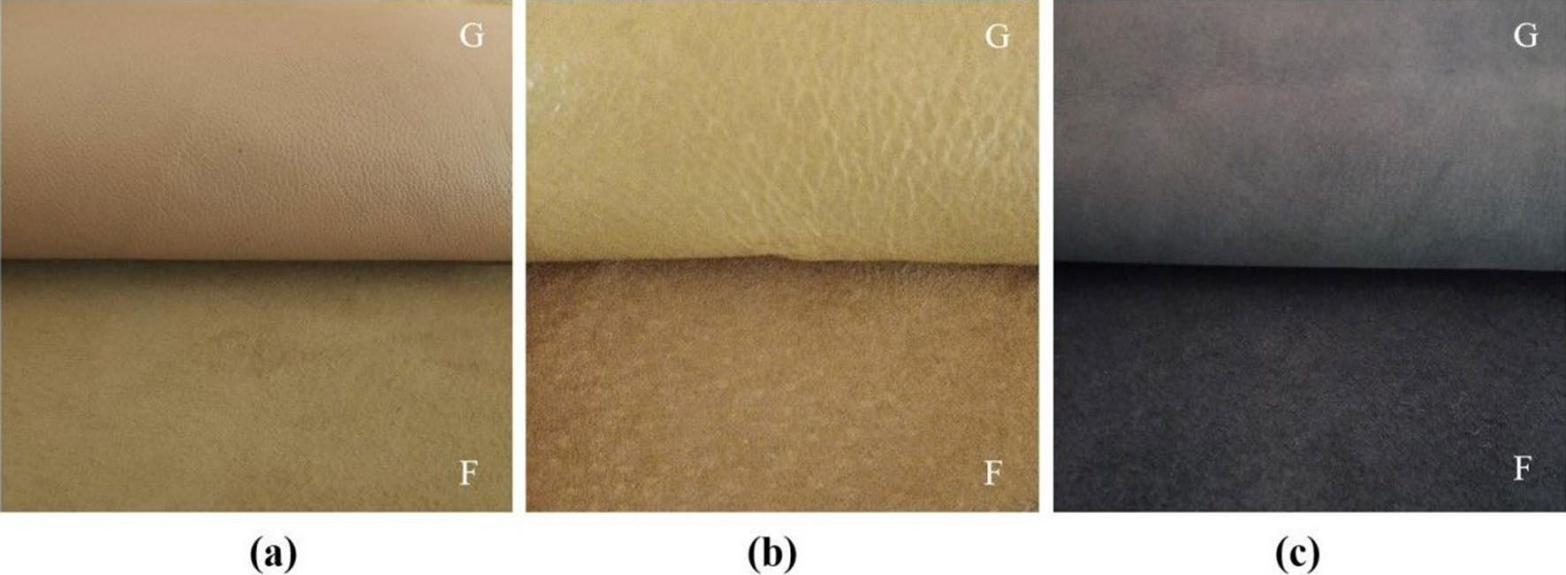
Tested material—leathers, for which samples A1 (**a**), A2 (**b**) and A3 (**c**) were collected; *G* grain side, *F* flesh side. Source: Author’s own research.

**Figure 2:**
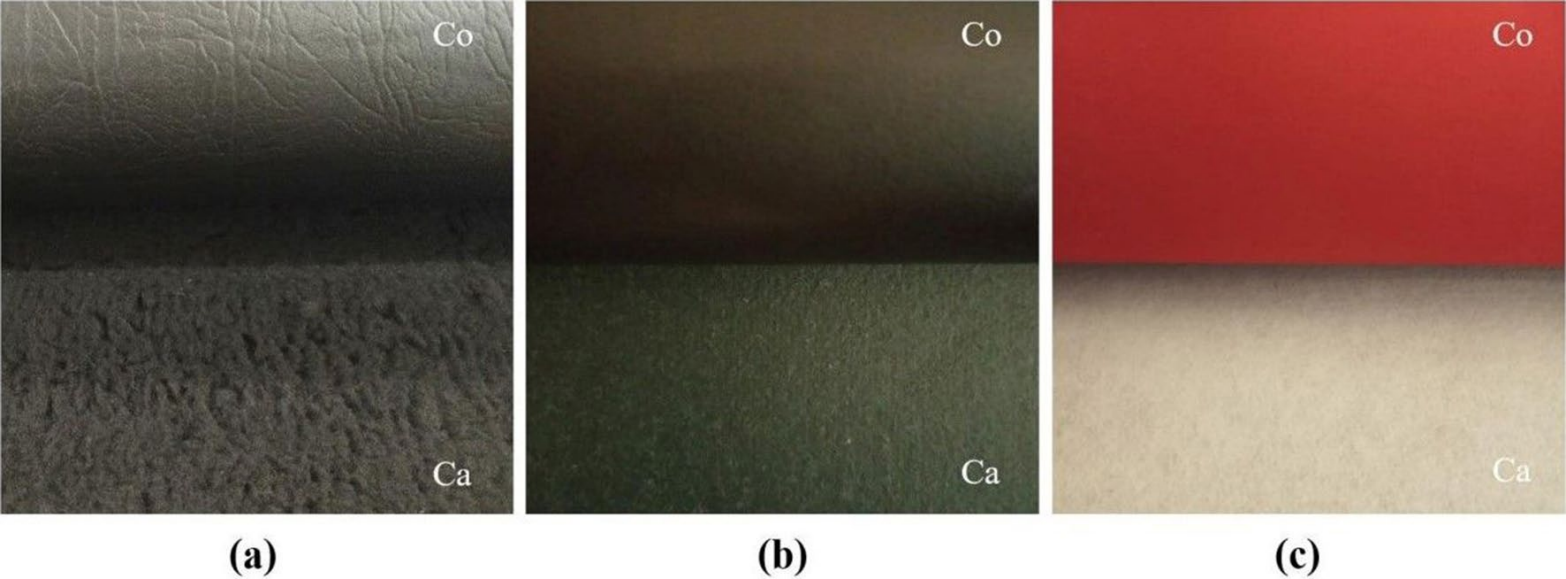
Tested material—artificial leather, for which samples B1 (**a**), B2 (**b**) and B3 (**c**) were collected; *Co* coating, *Ca* carrier. Source: Author’s own research.

**Figure 3:**
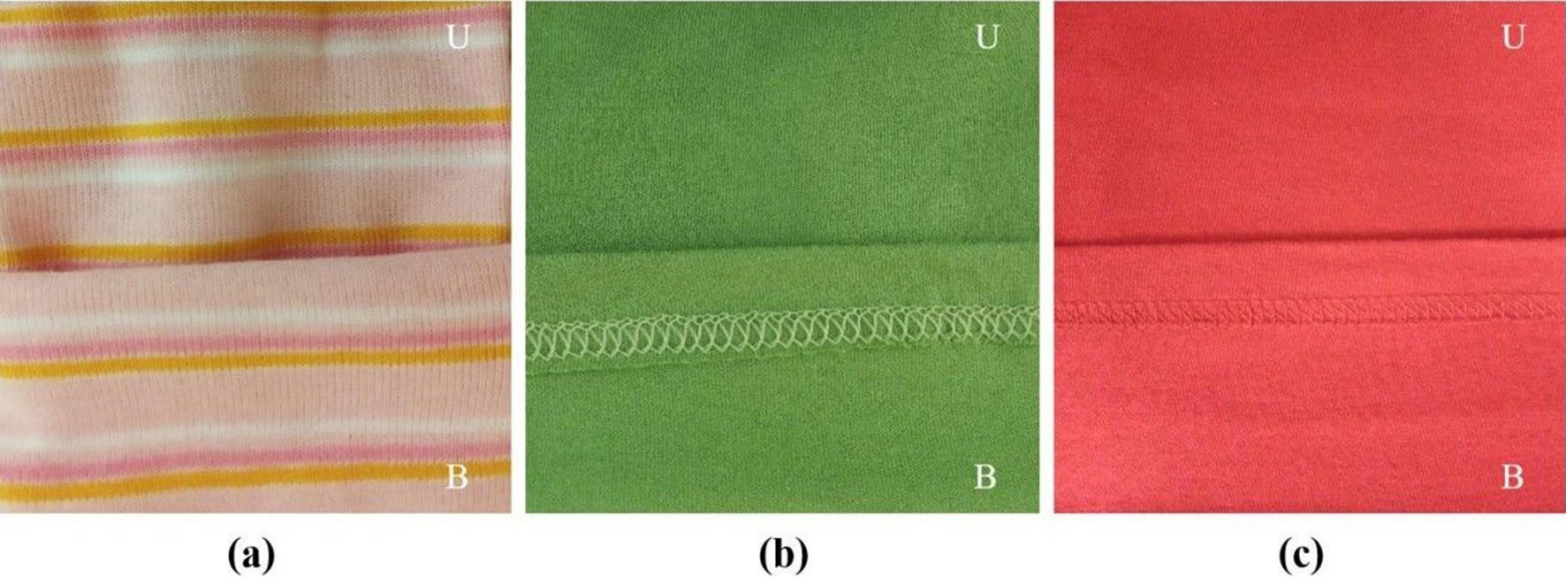
Tested material—textiles, for which samples C1 (**a**), C2 (**b**) and C3 (**c**) were collected; *U* upper, *B* bottom. Source: Author’s own research.

Nine materials were tested, the detailed characteristics of which are presented in Table 1. They included leathers, for which laboratory samples A1, A2 and A3 were collected (Fig. 1), artificial leathers, for which laboratory samples B1, B2 and B3 were collected (Fig. 2) and textiles, for which laboratory samples C1, C2 and C3 were collected (Fig. 3). All the materials tested were approved for marketing and available to consumers in the Polish market. Leathers were obtained from a tannery located in the south of Poland; artificial leathers were purchased from a domestic manufacturer of coated materials, while children’s clothes, from which samples C1–C3 were collected for testing, were purchased at a local flea market. According to the information provided on the tags, the products were manufactured in Turkey and Great Britain (Table 1). The materials for the tests were selected randomly considering the type of contact with the human body, i.e., direct or not direct, bearing in mind the possible presence of heavy metals in their extracts.

**Table 1:** Leathers, artificial leathers, and textiles. Characteristics of the tested material. Source: Author’s own research.

Sample	A1	A2	A3
**Leathers**			
Type of leather and method of finish^a^	Goat, aniline, soft, finished with a translucent layer, with a visible natural grain pattern, dyed pale pink	Pig, grain, soft, glossy, dyed beige	Cowhide, nubuck, delicately sanded grain, dyed purple
Method of tanning^a^	Chrome	Chrome	Chrome
Intended use^a^	Shoe lining	Shoe lining	Shoe uppers
Contact with the skin	Direct	Direct	Indirect
Country of origin	Poland	Poland	Poland

Sample	B1	B2	B3
**Artificial leather**			
Material characteristics^a^	Laminate composed of three layers: outer—knitted fabric coated with polyvinyl chloride, middle—polyurethane foam, and the inner layer constituting the research material—fur knit—acrylic fiber; black version	Synthetic poromeric ‘leather’: coagulated fabric coated with polyurethane; black version	Nubuck artificial, non woven fabric coated with polyvinyl chloride, red version
Intended use^a^	Insulated uppers of winter shoes	Shoe lining	Uppers of winter, summer and work shoes
Contact with the skin	Direct	Direct	Indirect
Country of origin	Poland	Poland	Poland

Sample	C1	C2	C3
**Textiles—children’s clothes**			
Characteristics of the product^b^	Baby bodysuit, 100% cotton, first quality, size 50/56, labelled with Öko-Tex Standard 100, pink color	T-shirt, 100% cotton, green color	Long-sleeve top, 100% cotton, for children aged 7–8, red color
Intended use of the product (child’s age)	Up to 36 months of age	Up to 36 months of age	Over 36 months (7–8 years^b^) of age
Contact with the skin	Direct	Direct	Direct
Country of origin^b^	Turkey	Turkey	Great Britain

^a^According to manufacturer’s declaration.

^b^According to the information on the tag.

### Testing methods

The determination of the selected heavy metals in the extracts obtained from the materials was conducted according to the method indicated in the documents *Testing Methods STANDARD 100 by OEKO-TEX*^35^ and *Testing Methods LEATHER STANDARD by OEKO-TEX*^36^. Following them, the tests were conducted in two stages. First, extraction of metals from leathers, artificial leathers and textiles was performed using an artificial acidic sweat solution. In the next step, quantitative determination of heavy metal content in the extracts was performed using atomic absorption spectrometry. AAS is one of the two alternative analytical techniques for determining the metal content in samples of materials, recommended by OEKO-TEX^35,36^ in addition to ICP. For the tests, the same procedure for sample preparation, metal extraction and metal determination with AAS was adopted for all the materials, i.e., leathers, artificial leathers, and textiles.

### Sampling, grinding, and drying of samples to a constant mass

Laboratory samples of leathers (A1, A2 and A3) were taken from butts following the guidelines of the ISO 2418 standard^37^, artificial leathers (B1, B2 and B3)—from the central part of a sample with an area of 200 × 150 cm, while textiles (C1, C2 and C3)—from the sleeves of children’s clothes (baby bodysuit, T-shirt and top). Next, the samples were ground in accordance with the guidelines of the ISO 4044 standard^38^ and placed in weighing vessels, dried and weighed with an accuracy of 0.001 g. The samples in the vessels were dried in a Memmert laboratory incubator at a temperature of 102 ± 2 °C, cooled in a desiccator and weighed until a constant mass was obtained^39^. The samples were dried for the purpose of standardisation, mainly because the water content in natural leather, and therefore its weight, depends on the ambient air humidity.

### Extraction of metals from the samples

**Figure 4:**
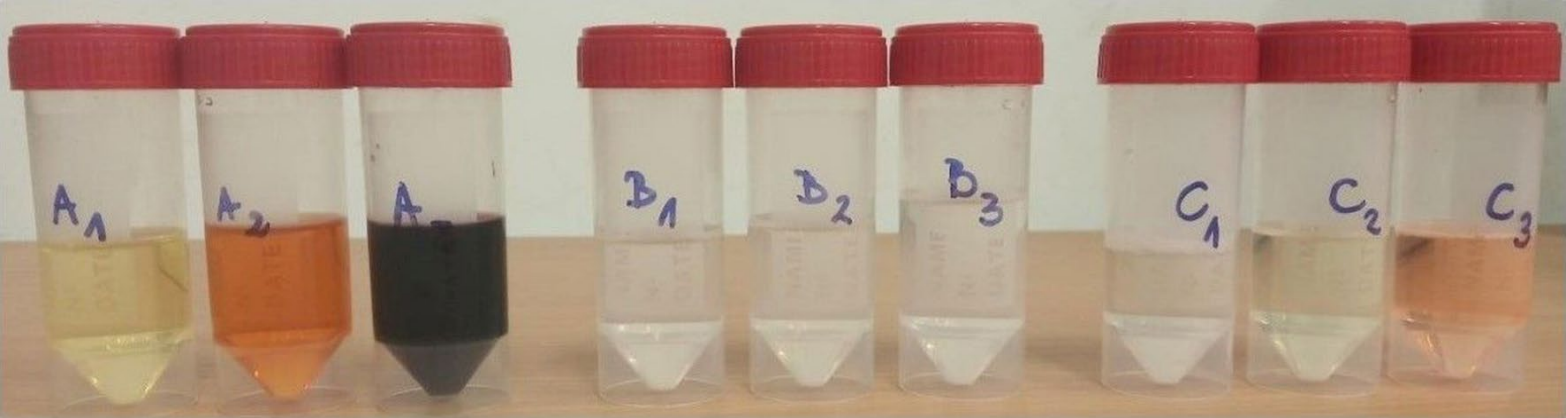
Solutions obtained after the extraction of heavy metals from the samples of leathers A1–A3, artificial leathers B1–B3 and textiles C1–C3. Source: Author’s own research.

Following the recommendations of OEKO-TEX^35,36^, an artificial acidic sweat solution was prepared based on the information contained in the ISO standards^40,41^. Both documents recommend the following composition (per litre): 0.5 g of l-histidine monohydrochloride monohydrate (C_6_H_9_N_3_O_2_⋯HCl⋯H_2_O), 5 g of sodium chloride (NaCl) and 2.2 g of sodium dihydrogen phosphate dihydrate (NaH_2_PO_4_⋯2H_2_O). The substances in the indicated amounts were dissolved in 1 L of demineralised water, and the resulting solution was adjusted to pH 5.5 (± 0.2) with a sodium hydroxide (NaOH) solution at a concentration of 0.1 mol/L. Then, approx. 1 g of the materials previously dried to a constant mass was weighed on an analytical balance and placed in Erlenmeyer flasks with a volume of 150 mL. In each flask, 50 mL of a freshly prepared artificial acidic sweat solution was added with a pipette. The solutions, together with materials, were shaken at a temperature of 37 ± 2 °C for 4 h and 5 min. After this time, the solutions were filtered through a paper filter (Whatman standard quality cellulose filter) with a pore size of 11 µm, and next through a nylon membrane filter (⌀25 mm) with a pore size of 0.45 µm^41^. The solutions obtained after the extraction are presented in Fig. 4. For the purpose of further analyses, the samples were acidified with nitric acid (HNO_3_).

### Determination of metal content in the extracts obtained from the tested materials with the use of AAS

The determination of the quantitative content of the selected heavy metals, i.e., As, Cd, Cr, Cu, Pb and Zn in the extracts obtained from leathers, artificial leathers and textiles was conducted in the Atomic Absorption Laboratory of the Laboratory at the Institute of Quality Sciences and Product Management. The determinations were made with the ICE 3000 ThermoScientific atomic absorption spectrometer, using the method of flame atomisation (for Cr, Cu and Zn) and atomisation in a graphite cuvette with background correction based on the Zeeman effect (for As, Cd and Pb). The flame atomisation technique enables achieving detection sensitivity of 0.05 ppm (mg/L) for Cr, 0.033 ppm (mg/L) for Cu and 0.01 ppm (mg/L) for Zn. The other aforementioned techniques enable achieving detection sensitivity of 5.2 ppb for As, 0.03 ppb for Cd and 0.12 ppb for Pb.

The actual tests were preceded by a preliminary determination of the number of elements analysed. It consisted of measuring the absorbance of the samples for radiation with a wavelength characteristic for the element being analysed without the use of standards. This allowed the estimation of metal concentrations and preparation of standard solutions. In order to check the purity of the reagents and the dilution water, a blank test was performed.

The analysis was performed on non-mineralised extracts, which after acidification with nitric acid (HNO_3_) were not diluted or diluted in such a way so that the concentration values of the elements examined were within the measuring range of the spectrometer. The determination of the content of Cd, Cu and Pb did not require dilution due to the presence of these metals in small quantities in the extracts from the samples. When measuring the content of Zn in the extracts obtained from samples B1, B2, B3, C1 and C2, five-fold dilution was used, while the analysis of the Cr content in the extracts from samples A1, A2 and A3 required ten-fold dilution. The research used standard wavelength lines (the most intense lines) for the individual elements. The determination of Cr content with the flame technique required the use of a mixture of acetylene (C_2_H_2_) with nitrous oxide (N_2_O), and in the case of Cu and Zn content—a mixture of acetylene (C_2_H_2_) and air was used. When determining the Cr content, nitrous oxide (N_2_O) was added in a minimum amount sufficient to obtain a flame with reducing properties. When measuring the Cu and Zn content, the gas mixture composition was stoichiometric, i.e., the gas proportions resulted from the stoichiometric ratio of the combustion reaction. During the Cr determination, the fuel flow rate was 4.3 L/min; for Cu, it was 1.1 L/min, and for Zn, it was 1.2 L/min.

The content of each element in the extract is presented as the so-callecd *corrected concentration.* This parameter considers the conversion factor that depends on the extract dilution. Moreover, it also considers the possible contamination of the reagents used with the element being analysed (blank test). The corrected concentration (c_corr_) is determined by the Eq. (1):

1 $${c}_{corr}=\frac{\left(c-{c}_{blank}\right){m}_{nomin}}{{m}_{sample}}{k}_{conver}{k}_{dilut,}$$

where c is the measured concentration, mg/dm^3^ or μg/dm^3^, c_blank_ is the concentration in a blank test, mg/dm^3^ or μg/dm^3^, m_nomin_ is the nominal mass (be default 1 g), m_sample_ is the sample mass, g, k_conver_ is the conversion factor, k_dilut_ is the dilution factor.

## Results and discussion

**Figure 5:**
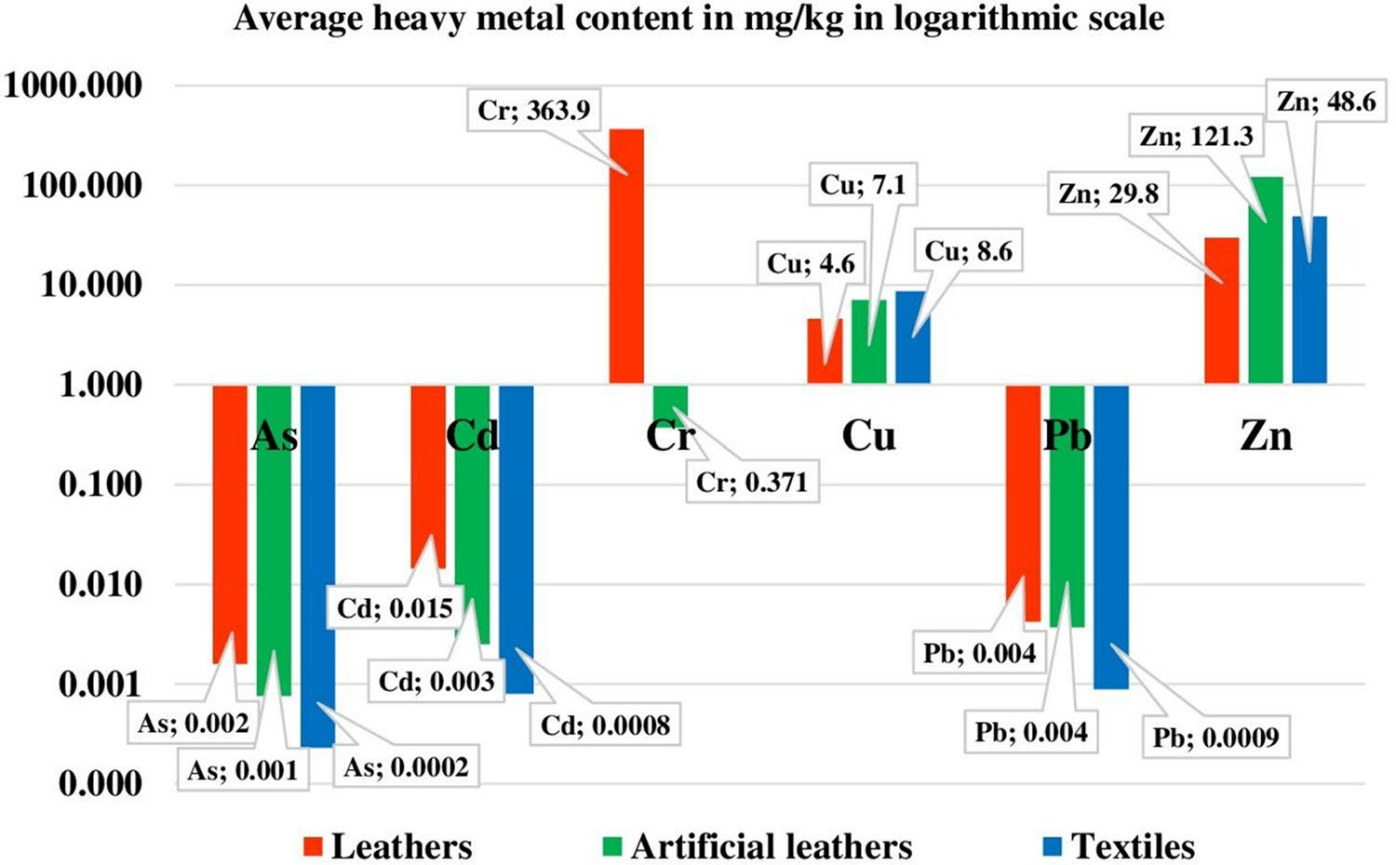
Average content of the tested metals in the extract from leathers, artificial leathers, and textiles. Source: Author’s own research.

The results of the study on the quantitative content of heavy metals As, Cd, Cr, Cu, Pb and Zn in the extracts obtained from leathers, artificial leathers, and textiles, rounded to significant figures, are presented in Tables 2, 3, 4. The values shown are the average of three measurements for each sample. The overall contents of As, Cd, Cr, Cu, Pb and Zn in the extracts obtained from the leathers, artificial leathers and textiles are shown in Fig. 5. Out of those aforementioned, the element present in the highest amounts in leather extracts was Cr (average content of approx. 364 mg/kg). In the case of extracts from artificial leathers and textiles, out of the metals analysed, Zn had the highest share (average content of approx. 121 mg/kg and approx. 49 mg/kg, respectively) (Fig. 5).

**Table 2:** As, Cd, Cr, Cu, Pb and Zn content in the extracts obtained from leathers and limits for their content according to OEKO-TEX and REACH regulation. Source: Author’s own research, limits of metal content based on the OEKO-TEX^34^—Annex IV and REACH Regulation^42^—Appendix 12.

Metal	Content detected in extracts from samples			Limit values according to OEKO-TEX^34^, less than		Concentration limit by weight^b^, according to REACH regulation^42^
	A1	A2	A3	Product class^a^		
				I	II, III, IV	
	mg/kg					
**Leathers**						
As	0.005	nd^c^	nd^c^	0.2	1.0	1.0
Cd	0.026	0.009	0.009	0.1		1.0
Cr	401.6	461.4	228.6	2.0	200.0	–
Cu	4.4	6.1	3.2	25.0	50.0	–
Pb	0.004	0.005	0.003	0.2	1.0	1.0
Zn	28.6	36.4	24.4	–	–	–

^a^I—products for infants and young children up to 36 months of age, II—products in direct contact with the skin, III—products in no direct contact with the skin, IV—decorative products; ^b^after extraction, expressed as As, Cd and Pb metal respectively, that can be extracted from the material; ^c^not detected.

**Table 3:** As, Cd, Cr, Cu, Pb and Zn content in the extracts obtained from artificial leathers and limits for their content according to OEKO-TEX and REACH regulation. Source: Author’s own research, limits of metal content based on the OEKO-TEX^33,34^—Annexes IV and REACH regulation^42^—Appendix 12.

Metal	Content detected in extracts from samples			Limit values according to OEKO-TEX^33,34^, less than		Concentration limit by weight^b^, according to REACH regulation^42^
	B1	B2	B3	Product class^a^		
				I	II, III, IV	
	mg/kg					
**Arificial leathers**						
As	nd^c^	nd^c^	0.002	0.2	1.0	1.0
Cd	0.003	0.004	0.001	0.1		1.0
Cr	1.1	nd^c^	nd^c^	1.0^d^/2.0^e^	2.0^d^/200.0^e^	–
Cu	10.7	6.0	4.6	25.0	50.0	–
Pb	0.006	0.003	0.003	0.2	1.0	1.0
Zn	103.6	87.6	172.7	–	–	–

^a^I—products for infants and young children up to 36 months of age, II—products in direct contact with the skin, III—products in no direct contact with the skin, IV—decorative products; ^b^after extraction, expressed as As, Cd and Pb metal respectively, that can be extracted from the material; ^c^not detected; ^d^limit according to OEKO-TEX^33^; ^e^limit according to OEKO-TEX^34^.

**Table 4:** As, Cd, Cr, Cu, Pb and Zn content in the extracts obtained from textiles and limits for their content according to OEKO-TEX and REACH Regulation. Source: Author’s own research, limits of metal content based on the OEKO-TEX^33^—Annex IV and REACH Regulation^42^—Appendix 12.

Metal	Content detected in extracts from samples			Limit values according to OEKO-TEX^33^, less than		Concentration limit by weight^b^, according to REACH regulation^42^
	C1	C2	C3	Product class^a^		
				I	II, III, IV	
	mg/kg					
**Textiles**						
As	nd^c^	nd^c^	0.001	0.2	1.0	1.0
Cd	nd^c^	0.002	0.001	0.1		1.0
Cr	nd^c^	nd^c^	nd^c^	1.0	2.0	–
Cu	5.7	15.5	4.6	25.0	50.0	–
Pb	0.003	nd^c^	nd^c^	0.2	1.0	1.0
Zn	65.7	59.3	20.8	–	–	–

^a^I—products for infants and young children up to 36 months of age, II—products in direct contact with the skin, III—products in no direct contact with the skin, IV—decorative products; ^b^after extraction, expressed as As, Cd and Pb metal respectively, that can be extracted from the material; ^c^not detected.

For comparison purposes, Tables 2, 3, 4 also include the limits for heavy metals extracted from products for infants and children up to 36 months of age, both with and without direct contact with the human body, as well as decorative products (product class I, II, III and IV, respectively), as recommended by the *STANDARD 100 by OEKO-TEX*^33^ and the *LEATHER STANDARD by OEKO-TEX*^34^. Annexe IV to the indicated standards includes the requirements for limit values for As, Cd, Cr, Cu and Pb. The STANDARD 100 by OEKO-TEX and the LEATHER STANDARD by OEKO-TEX marks are awarded to textiles, leathers and leather products that meet the requirements for the content of harmful substances specified in the aforementioned OEKO-TEX standards^33,34^. Thus, the voluntary submission of the product to the certification process, its successful completion and obtaining any of the above-mentioned marks prove the high quality of a product as understood in the context of its safety for the consumer. Furthermore, Tables 2, 3, 4 also includes maximum permitted contents of As, Cd and Pb according to Regulation (EC) No 1907/2006 of the European Parliament and of the Council of 18 December 2006 concerning the Registration, Evaluation, Authorisation and Restriction of Chemicals (REACH)^42^. This regulation was designed to ensure a high level of protection of both human health and the environment. It establishes rules concerning, among others, the manufacturing, placing on the market and use of substances in articles^42^.

### Analysis of metal contents in extracts from natural leathers

As suggested by the data presented in Table 2, the element present in the smallest amount in the extract obtained from sample A1 was Pb (0.004 mg/kg). In the case of samples A2 and A3, no As was found at all, while the element detected in the smallest amounts was also Pb (0.005 mg/kg and 0.003 mg/kg, respectively). When taking into account the heavy metals analysed, all the extracts were characterised by the highest content of Cr (lining leathers—more than 401 mg/kg, upper leather—more than 228 mg/kg). Significantly lower contents of this metal in extracts obtained using artificial sweat from insole leathers (53.30 mg/kg) and upper leathers (67.18 mg/kg) were detected by ICP-AES by Basaran et al.^27^. They also determined a lower content of Zn in the samples (insole leathers—9.01 mg/kg, upper leathers—2.26 mg/kg). Turkish insole leathers contained less Cu (3.92 mg/kg) as compared with its content in the extracts from the tested samples of lining leathers A1—4.4 mg/kg and A2—6.1 mg/kg (Table 2). A similar pattern was observed for upper leather (Turkish leathers—2.14 mg/kg, sample A3—3.2 mg/kg). However, leathers for both inner and outer footwear components tested by Basaran et al.^27^ contained more Cd (0.16 mg/kg and 0.10 mg/kg, respectively) and Pb (2.32 mg/kg and 0.78 mg/kg, respectively) compared with the leathers tested in Poland (Table 2).

Markedly higher contents of As, Cd, Cr and Pb, i.e., at the average levels of 0.63 mg/kg, 1.28 mg/kg, 29,613.75 mg/kg and 1.21 mg/kg, respectively, were demonstrated by Karavana et al.^26^ who used the ICP-OES technique to examine solutions extracted from chrome leathers for children’s shoes treated with HCl solution. No Cd was detected in extracts from lining and upper leathers prepared by Yaşa et al.^25^ using artificial sweat; further to this, lower contents of Cr (2.95–14.10 mg/kg and 2.82–7.74 mg/kg, respectively) and Zn (0.00–15.86 mg/kg and 0.00–0.00 mg/kg, respectively) were found compared with samples A1–A3 (Table 2). Cu was present in the lining leather samples A1 and A2 at 4.4 mg/kg and 6.1 mg/kg, respectively, while in the lining leather samples tested in Turkey, its content was lower and ranged from 0.00 to 0.07 mg/kg. In upper leather (sample A3), Cu was detected in amount of 3.2 mg/kg, in Turkish leathers in a range from 0.00 to 12.58 mg/kg. Studies by Yaşa et al.^25^ using ICP-OES showed an average higher Pb content in leathers (values in the range of 0.00–0.29 mg/kg for lining leathers and 0.00–0.02 mg/kg for upper leathers, respectively) compared with samples tested in Poland (Table 2).

Extracts from natural leathers prepared in artificial sweat solution studied by Aslan and Üzüm^24^, unlike samples A1–A3 (Table 2), did not contain Cd and Pb at all. The mean Cu content detected in them at 6.57 mg/kg was higher compared with the Cu content in samples A1 (4.4 mg/kg), A2 (6.1 mg/kg) and A3 (3.2 mg/kg). The opposite pattern was observed for Cr and Zn. Studies using ICP-OES showed that the leathers analysed by Aslan and Üzüm^24^ contained significantly less Cr (168.42 mg/kg) and Zn (1.23 mg/kg) than the samples tested in Poland (Table 2).

The high chromium content detected in the extracts obtained from the lining and upper leathers (Table 2) is due to the tanning method. As shown in Table 1, all the leathers tested were chromium tanned (i.e., using chromium (III) salts). This pattern was also confirmed in earlier studies^24–27^. The presence of the other metals analysed, i.e. As, Cd, Cu, Pb and Zn, detected in much smaller amounts compared with Cr, should be linked to the chemical agents used during leather processing at the tannery, i.e., dyeing and finishing^24–27^.

When comparing the results concerning the content of heavy metals in the extracts obtained from leather samples with the limits set out by the *LEATHER STANDARD by OEKO-TEX*—Annex IV^34^, it can be concluded that they all meet the requirements for the content of As, Cd, Cu, Pb, while a heavy metal that was present in too large amounts in the extracts from the samples was Cr. None of the leathers met the stringent requirements for children's products (< 2.0 mg/kg), for products with and without direct contact with the skin, or decorative products (< 200 mg/kg). In the extracts of lining leathers (A1 and A2), and thus the ones that may have direct contact with the user's body, the Cr content was more than double the concentration limit (Table 2). The analyses also confirmed that As, Cd, and Pb contents in the extracts obtained from the leather samples were much lower than the concentration limit (1 mg/kg) recommended by the REACH Regulation^42^ (Table 2).

### Analysis of metal contents in extracts from artificial leathers

In the extracts from the samples of artificial leathers marked as B1 and B2, As was not detected, and no Cr was found in the extract from sample B2 or in the extract from sample B3 (Table 3). In the materials tested, the lowest amounts among the heavy metals identified were found for Cd (extract from sample B1—0.003 mg/kg and extract from sample B3—0.001 mg/kg) and Pb (extract from sample B2—0.003 mg/kg). The tests with the use of AAS confirmed the highest content of Zn in extracts from samples B1, B2 and B3.

In 2015, Aslan and Üzüm^24^ investigated heavy metal contents in artificial leather extracts prepared in an artificial sweat solution using ICP-ORS. During their analyses, they did not detect the presence of Cd and Pb in the samples, while the Cu contents, which they tested at 0.03 mg/kg, and Zn contents at 0.01 mg/kg, were significantly lower compared with those obtained for samples B1, B2 and B3 (Table 3), especially for Zn. The only element determined by the Turkish researchers in higher amounts compared to the samples tested in Poland was Cr (11.48 mg/kg). The scientists concluded that the contents of heavy metals in artificial leather are much lower compared with their contents in the natural leathers that they also tested. According to them, the presence of heavy metals in artificial leather is related to the use of chemicals, dyes, and pigments during the production process. It may also be due to contaminants of different origins^24^.

A high content of Zn in the samples of artificial leathers B1 (103.6 mg/kg), B2 (87.6 mg/kg) and B3 (172.7 mg/kg) should be explained by the fact that compounds of this metal are used in the production of these materials because of their specific properties. According to Ranta-Korpi et al.^43^, Zn is the most commonly used transition metal in plastics. Its compounds act as thermal stabilisers (PVC production), antimicrobial agents (PVC, PU production), flame retardants, and pigments. An example of such a pigment is zinc sulphide, used for plastics and also for leathers^44^. Zinc is a micromineral that plays an important role in the human body, unlike Cd, Pb or As, the contents of which in materials is permitted in low amounts (Table 3), which is related to the mechanisms of their toxic effects^45^. For example, Zn is responsible for the normal development of a fetus, influences the functioning of the immune system and wound healing; it is also a component of enzymes, a metal necessary to produce protein and genetic material^46^.

The results obtained for artificial leathers were analysed in terms of the requirements contained in the *STANDARD 100 by OEKO-TEX*—Annex IV^33^ concerning textile products, as well as in relation to the limits set out in the *LEATHER STANDARD by OEKO-TEX*—Annex IV^34^ intended for leathers and leather products. It was found that all the artificial leathers in the test meet the requirements of both standards regarding the content of metals such as As, Cd, Cu and Pb in their extracts, both taking into account the limits for products intended for children, those that are in direct and not direct contact with the skin, as well as decorative ones. In the case of the extract from sample B1, the Cr content detected at the level of 1.1 mg/kg exceeds the permissible limit (< 1.0 mg/kg) of the *STANDARD 100 by OEKO-TEX*^33^ specified for products of class I. When considering the identified content of this element in the extract from sample B1 in accordance with the guidelines of the *LEATHER STANDARD by OEKO-TEX*^34^, it can be concluded that the material tested contains low amounts of Cr (Table 3). In the extracts from artificial leathers, the detected contents of As, Cd and Pb (Table 3) met the guidelines of the REACH Regulation^42^ and were well below the limit of 1 mg/kg as in the case of natural leather.

### Analysis of metal contents in extracts from textiles

No Cr was detected in any of the extracts obtained from textiles (C1–C3); moreover, As was not found in the extracts from samples C1 and C2, in the extract from sample C1—there was no Cd, and from samples C2 and C3—no Pb was found (Table 4). In the extract obtained from sample C1, collected from a baby bodysuit, the smallest amount (0.003 mg/kg) out of the metals tested was found for Pb. Regarding sample C2 (children's T-shirt), the metal with the least amount detected in the extract was Cd (0.002 mg/kg). The extract obtained from sample C3 collected from the children's top was characterised by the lowest content of As and Cd; each of these elements was detected in the amount of 0.001 mg/kg. The heavy metal present in the highest amount in the extracts obtained from the materials tested was Zn.

In 2004, Rybicki et al.^32^ carried out analyses of heavy metal contents in extracts from cotton bed cloth, obtained using artificial sweat. They did not detect Cd and Pb in the textiles tested. The first of the metals were present in two samples analysed in the experiment, i.e., in sample C2 (at the level of 0.002 mg/kg) and sample C3 (at the level of 0.001 mg/kg), whereas Pb was detected only in sample C1 (in the amount of 0.003 mg/kg)—Table 4. The ICP method used in 2004 allowed the researchers to identify Zn (11.35 mg/kg) in the materials in lower amounts compared with the results obtained for the children’s clothes (samples C1, C2 and C3—Table 4). However, the opposite pattern was observed for Cu, which was detected at the level of 279.7 mg/kg in cotton bed cloth, while the content of this metal in children’s clothes ranged from 4.6 mg/kg (sample C3) to 15.5 mg/kg (sample C2). Furthermore, Cr was not detected in any of the extracts extracted from the clothes (Table 4), while in the study by Rybicki et al.^32^, it was determined in cotton bed cloth in the amount of 5.34 mg/kg.

In Turkey, Cr was also detected at 0.11 mg/kg by the MP-AES method in extracts from cotton textiles fibres prepared with artificial sweat^29^. Apart from this metal, Cu (3.16 mg/kg) and Pb (1.57 mg/kg) contents were determined in the textile samples. The content of the former metal was lower compared with the results obtained for all the children’s clothes (sample C1—5.7 mg/kg, sample C2—15.5 mg/kg, C3—4.6 mg/kg). However, Pb was only detected in sample C1—a baby bodysuit, in the amount of 0.003 mg/kg (Table 4).

Chrome content in extracts from textiles prepared by using artificial sweat was studied by Tonetti and Innocenti^30^ using AAS. In the material dyed using a higher concentration of chromium dye, this metal was detected in the range between 97.80 mg/kg and 134.32 mg/kg depending on the specific process parameters. On the other hand, in the material where a lower concentration of dye was used, the Cr content ranged from 6.09 to 10.92 mg/kg.

The AAS method was used by researchers from Turkey to determine the contents of selected heavy metals in textile samples following microwave digestion^31^. Cadmium was detected by them in the range of 0.10–0.25 mg/kg. This amount was significantly higher compared with the results obtained for the extracts from C2 children’s clothes, i.e., 0.002 mg/kg and C3—0.001 mg/kg (Table 4). The Turkish researchers identified Cu in the textiles in the range of 0.76–341 mg/kg. By comparison, the highest detected content of this metal in the children’s clothes was at the level of 15.5 mg/kg (sample C2). The content of Zn in the case of the Turkish textiles ranged from 0.63 to 4.84 mg/kg^31^, whereas in the samples tested in Poland, it ranged from 20.8 mg/kg (sample C3) to 65.7 mg/kg (sample C1).

The presence of Zn in the materials tested (Table 4) should be explained by the use of its compounds during production processes. By way of example, in the textile industry, zinc oxide (ZnO) acts as a filler and also makes it possible to give a white colour and elasticity to the material. It is also used because of its antimicrobial properties^47^.

As can be seen in the list in Table 4, the textiles tested (samples C1–C3) meet the requirements of the *STANDARD 100 by OEKO-TEX***—**Annex IV^33^ in terms of the permissible content of heavy metals in their extracts applicable to children's products (product class I) as well as the remaining products (product class II–IV). In the extracts obtained from children’s clothes, the limit for the content of As, Cd and Pb was not exceeded in relation to the REACH Regulation guidelines^42^ (Table 4).

### Analysis of metal contents in extracts from the materials tested in terms of the requirements of STANDARD 100 by OEKO-TEX: Annex VI

The only metal for which no requirements in Annexes IV to *STANDARD 100 by OEKO-TEX*^33^ and the *LEATHER STANDARD by OEKO-TEX*^34^ are provided is Zn. The limits for all the elements tested, including Zn, can be found in the *STANDARD 100 by OEKO-TEX*^33^ applicable to textiles—Annex VI. This Annex sets out stricter guidelines and is intended for companies that support the Detox campaign and want or must (due to the specific requirements of their customers) follow the requirements thereof. This campaign was launched in 2011 by Greenpeace to draw attention to the impact of the world’s biggest clothing brands and their suppliers on water pollution caused by toxic chemicals from the textile industry. The actions included inspections in the clothes-producing countries and testing clothes for harmful chemicals. The campaign focused on eliminating the release of harmful chemicals from the supply chain and from the products themselves. This challenge was taken up by many international clothing brands, including Nike, Zara, Benetton, Burberry, and Victoria’s Secret^48^. As Ortega-Egea and García-de-Frutos^49^ point out, the Detox campaign was a response to the need for sustainable changes in the textile industry. However, despite the many actions taken in the textile and clothing industry towards sustainability, the topic still needs attention, especially in the area of consumers’ pro-environmental behaviour^50^.

Considering the extended requirements contained in Annex VI to the *STANDARD 100 by OEKO-TEX*^33^, the permissible content of the tested metals in the extracts obtained from materials should be lower than, for:

- As: 0.2 mg/kg (product class I–IV),
- Cd: 0.1 mg/kg (product class I–IV),
- Cr: 1.0 mg/kg (product class I–IV),
- Cu: 25 mg/kg (product class I) or 50 mg/kg (product class II–IV),
- Pb: 0.2 mg/kg (product class I–IV),
- Zn: 750 mg/kg (product class I–IV).

Comparing the obtained results of the content of the metals in the extracts of the tested materials (Tables 2, 3, 4) with the limits set out in Annex VI^22^, it can be concluded that the requirements for As, Cd, Cu and Pb, as well as Zn were met. In all the leather extracts (samples A1–A3), the permissible Cr content was exceeded, similarly to one extract from an artificial leather (sample B1). However, according to the *STANDARD 100 by OEKO-TEX*^33^, in the case of leathers and leather accessories, the guidelines specified in the *LEATHER STANDARD by OEKO-TEX*^34^ should be applied.

## Summary and conclusions

The study conducted with the use of atomic absorption spectrometry made it possible to quantify the content of heavy metals, i.e. chromium, copper, zinc (flame atomisation), arsenic, cadmium and lead (atomisation in a graphite cuvette) in extracts from leathers and artificial leathers used in the production of uppers and inside components of footwear, as well as in extracts from ready-made textile products, e.g., children's clothes.

The extracts obtained from the tested textiles and the vast majority of artificial leathers contain heavy metals in the amounts permissible by OEKO-TEX, considered safe for consumers. In the case of one item of artificial leather, i.e., acrylic knit fur, a chromium content of 1.1 mg/kg was too high compared to the limit (less than 1.0 mg/kg) specified in the *STANDARD 100 by OEKO-TEX* for children's products. This proves the high quality of materials and products of this type available on the market, especially considering the fact that the requirements indicated by OEKO-TEX are often more restrictive than others, whether domestic or international.

In the case of the tested leathers, their quality may raise concerns due to their non-compliance with the OEKO-TEX requirements regarding the chromium content in the extracts obtained from them. This metal was detected in the samples at the levels of 401.6 mg/kg, 461.4 mg/kg and 228.6 mg/kg, respectively, where the maximum permissible values are less than 2.0 mg/kg for children’s products and less than 200 mg/kg for products that come in direct or indirect contact with the skin. The high chromium content in extracts from leathers purchased from a national tannery may be due to the low level of washing after retanning.

Materials that are approved for marketing may have elevated heavy metal contents, as has been demonstrated by the example of chromium. Bearing in mind the fact that the presence of heavy metals in too large amounts, especially in products intended for children and those that will come in close contact with the skin is a serious problem due to their irritant, allergic and toxic effects, measures should be taken aimed at detecting them more effectively and eliminating them from the markets. For example, this can be achieved by stepping up checks on materials at the production stage and on products approved for marketing. Another solution to the problem may be by reducing the use of chemicals containing harmful metals in the production processes or replacing them with other, more ecological ones, which is a great challenge for the clothing and footwear industry.

The results obtained confirm that it is correct and necessary to continue the research on the materials available in the global markets, not only in the context of heavy metals but also a wider range of harmful chemical substances that may pose a threat to consumer health and the environment.

## Data Availability

The datasets generated and analysed during the current study are available from the corresponding author (Elżbieta Bielak) on reasonable request.
